# Emerging Paradigms in Internal Root Resorption Management: Harnessing the Power of Bioceramics

**DOI:** 10.7759/cureus.45149

**Published:** 2023-09-13

**Authors:** Jay Bhopatkar, Anuja Ikhar, Pradnya Nikhade, Manoj Chandak, Paridhi Agrawal

**Affiliations:** 1 Department of Conservative Dentistry and Endodontics, Sharad Pawar Dental College and Hospital, Datta Meghe Institute of Higher Education and Research, Wardha, IND

**Keywords:** diode laser therapy, extensive idiopathic perforating internal root resorption, non-surgical treatment, bio-c repair, bioceramics

## Abstract

Internal root resorption is a pathological activity involving dentin deterioration within the root canal walls. Numerous variables, including traumatic injury, infection, and orthodontic therapy, can trigger this process. Traditional materials such as mineral trioxide aggregate (MTA) have been utilized to treat internal root resorption but have limitations such as tooth discoloration and handling challenges. Bioceramic materials, such as Bio-C Repair, have emerged as possible MTA substitutes. This case study outlines the effective management of idiopathic extensive perforating internal root resorption using a non-surgical laser-assisted approach and the application of Bio-C Repair as an obturation material. The treatment resulted in the resolution of symptoms and the restoration of periapical tissues. Bioceramics, with their unique composition and favorable biological properties, offer the potential for effective tissue repair and provide alternatives to traditional materials in the treatment of internal resorption. The utilization of bioceramics, including Bio-C Repair, holds promise for achieving successful outcomes and preserving natural dentition.

## Introduction

Resorption refers to the loss of dentin, cementum, or bone and can occur as an outcome of physiological or pathological processes [1]. External (replacement, inflammatory, surface) and internal (replacement, inflammatory) categories have been used to classify tooth resorption [2]. The middle and apical third of the root canal wall’s dentin and dentinal tubules are gradually destroyed during internal root resorption [3]. Orthodontic therapy, infection, and traumatic injury are among the suggested etiological factors for internal root resorption [4]. The event of resorption involves the breakdown of the organic framework and the subsequent disintegration of the inorganic mineral form [5].

Clinically, internal resorption is often silent, although a reddish area (pink spot) may be present, suggesting granulation tissue that is visible through the region of resorption. Radiographs are essential for the diagnosis of this condition, revealing a radiolucent pulp space expansion that is spherical to oval, with smooth, well-defined edges and disruption of the original root canal pattern [2,4]. Visual examination based on variations in the color of the tooth crown, radiographic diagnostic tools such as conventional and cone-beam computed tomography (CBCT), as well as light microscopy and electron microscopy, are used to identify internal resorption [6,7].

Several materials can be used to treat internal root resorption, including mineral trioxide aggregate (MTA), hydrophilic plastic polymers, Biodentin, Super EBA, zinc oxide eugenol, glass ionomer cement, zinc acetate cement, composite resin, thermoplasticized gutta-percha, and amalgam alloy. MTA is often used due to its sealing characteristics, biocompatibility, and capability to cause cementogenesis and osteogenesis [8,9]. However, there are drawbacks to MTA, including tooth discoloration, handling challenges, a lengthy setting period, and the discharge of heavy metals.

To address these limitations, a new category of materials called bioceramics has emerged. Bioceramics comprise calcium phosphate, calcium silicates, calcium hydroxide, tantalum oxide, zirconium oxide, thickeners, and putties [10]. These materials offer improved handling and setting properties, high pH, potential bioactivity, chemical stability, good radiopacity, increased root fracture resistance, and resistance to resorption. Additionally, they interact with periapical tissue stem cells to encourage biological sealing and trigger the healing process [11].

One such bioceramic material is Bio-C Repair, a ready-to-use material introduced in endodontics. It offers handling and insertion advantages and has comparable biomineralization properties, biocompatibility, and cytotoxicity to MTA-high plasticity and white MTA [12]. Bio-C Repair exhibits identical pH levels to MTA but demonstrates a higher cellular survival rate and adhesion, demonstrating greater bioactivity. When combined with a chemical approach, this substance exhibits high cytocompatibility, comparable to Biodentine and MTA, and has a promising ultrastructural morphology on human dental pulp cells [13].

## Case presentation

A 55-year-old male patient presented to the Department of Conservative Dentistry and Endodontics with a chief complaint of severe pain in the lower front region of the jaw. The pain had been ongoing for two weeks. The patient had a history of root canal treatment for teeth #31 and #41 performed two years ago. There was no history of traumatic injury or orthodontic therapy, and the patient’s medical history was not relevant to the current complaint.

**Figure 1:**
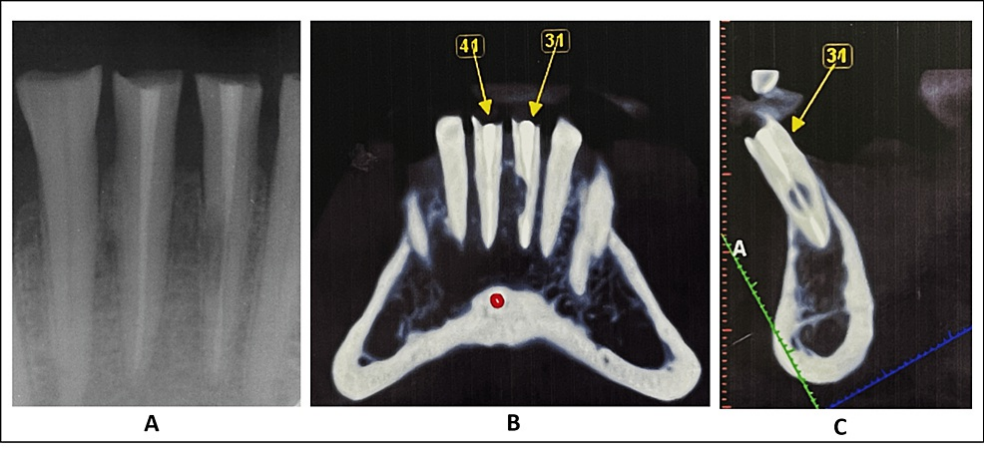
Preoperative assessment. A: Preoperative intraoral periapical radiograph. B: Cone-beam computed tomography slice of teeth #31 and #41. C: Sagittal view of internal root resorption in tooth #31.

Upon clinical investigation, discolored composite restorations with intact margins were observed in teeth #31 and #41. The probing depth for both teeth was within the acceptable range. However, both teeth were tender upon palpation and percussion, with tooth #31 producing a high-pitched metallic sound suggestive of root canal defect such as root resorption. Neither tooth #31 nor #41 exhibited mobility. In tooth #31, the apical region showed soft enlargement of the buccal tissues. A periapical radiographic examination revealed radio-opaque shadow suggestive of gutta-percha filling in both teeth, while tooth #31 displayed a significant and distinct oval radiolucency in the middle third of the root canal, with only a tiny portion of the apex remaining intact (Figure 1, Panel A).

Considering the clinical and radiological observations, the diagnosis was determined as persistent apical periodontitis for tooth #41 and idiopathic extensive perforating internal root resorption for tooth #31. To further assess the resorptive lesion, limited CBCT using the Planmeca Promax 3D machine (Planmeca, Finland) with a field of view of 80 × 80 mm and slice thickness of 1 mm was employed. The CBCT scan revealed that the internal root resorption in tooth #31 was substantial and had penetrated the middle third of the canal towards the mesial aspect, resulting in bone perforation. This evaluation was conducted by analyzing the coronal sagittal and axial slices of the CBCT scans (Figure 1, Panels B and C).

The clinical observations and treatment options were discussed with the patient, taking into consideration the patient’s anxiety and reluctance to undergo surgical procedures or extractions. As a result, a decision was made to pursue non-surgical laser-assisted management for the progressive perforating resorption in tooth #31, while tooth #41 would undergo conventional retreatment. This treatment plan was chosen to accommodate the patient’s preferences and minimize the need for invasive procedures.

**Figure 2:**
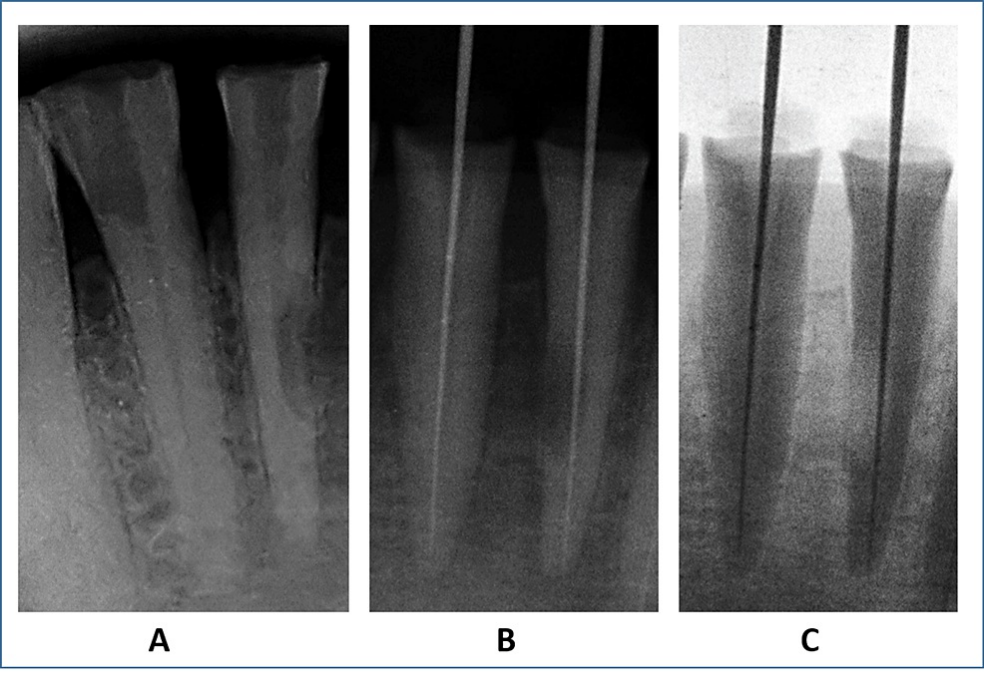
Intraoperative assessment. A: Post-gutta-percha removal radiograph. B: Working length radiograph. C: Working length radiograph inverse.

During the first visit, rubber dam isolation was achived using Optra Dam (Coltene Whaldent, Ohio, USA). Subsequently, the root canals of both teeth were examined using a surgical microscope (Labomed Prima DNT, Labomed, USA). The previous gutta-percha filling was successfully removed (Figure 2A). In tooth #31, radiographic working length determination was performed due to the presence of granulation tissue and excessive bleeding. An electronic apex locator (Root ZX Mini, J Morita, Japan) was used for tooth #41, and the working length was re-confirmed radiographically (Figures 2B, 2C). The presence of granulation tissue in tooth #31 required manual debridement of the visible tissue. Subsequently, biomechanical preparation was carried out up to 26-04% using Trunatomy files (Dentsply Sirona, USA), and hemostasis was achieved by irrigating with 5% sodium hypochlorite (Parcan, Septodont, India). In tooth #41, conventional biomechanical preparation was performed up to 26-04% using Trunatomy files (Dentsply Sirona, USA). Temporary calcium hydroxide (RC Cal, Prime Dental, India) dressing was placed in both teeth, and the patient was scheduled for a follow-up appointment after seven days.

During the second visit, a precautionary triple antibiotic temporary dressing (Neotemp, Neoendo, India) was applied to both teeth. This was done to minimize the risk of infection. The patient was then re-scheduled for a follow-up evaluation after 10 days.

**Figure 3:**
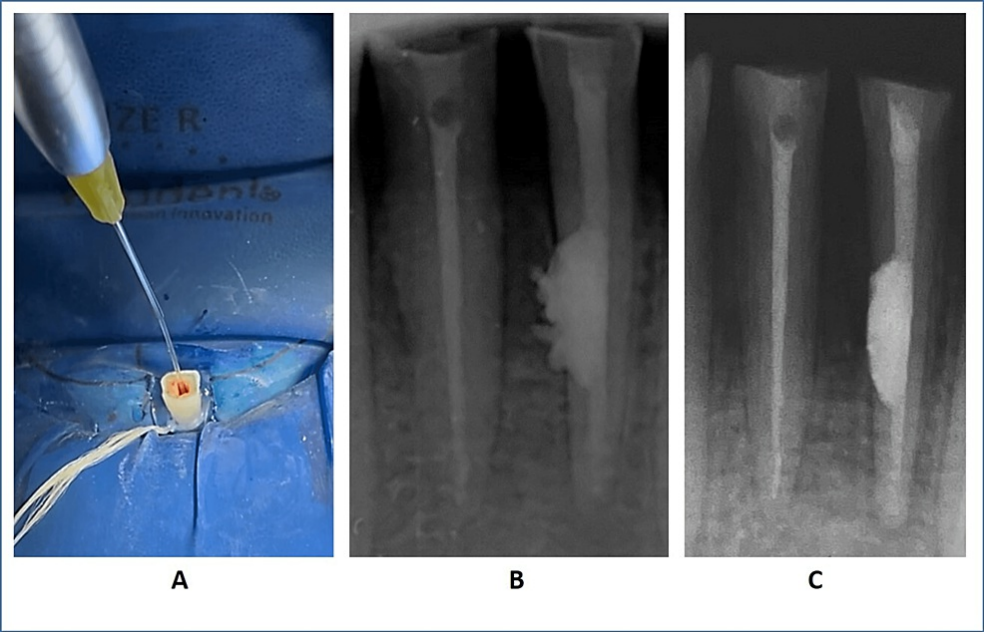
Postoperative assessment with a follow-up radiograph. A: Laser debridement. B: Immediate postoperative radiograph; C: Six-month follow-up radiograph.

During the third visit, the patient remained asymptomatic. Upon removing the temporary dressing (Neotemp, Neoendo, India) in tooth #31, small bits of granulation tissue were observed along with bleeding. To accurately assess the perforation level, CBCT scans measurements were performed. The scans indicated a perforation length of 8 mm. To address the granulation tissue, 90% trichloroacetic acid was used for debridement, but due to its caustic effect, it is highly contraindicated in cases with extensive perforating internal root resorption; hence, diode laser-assisted granulation tissue debridement (Biolase epic-x, Biolase, India) was performed in tooth #31 (Figure 3, Panel A). The procedure was performed under a surgical microscope (Labomed Prima DNT, Labomed, USA), and the laser tip was marked for 8 mm. After this step, copious irrigation with 5% sodium hypochlorite (Parcan, Septodont, India) was performed in both teeth. Following irrigation, both canals were dried using paper points. At this stage, both canals were found to be dry and free of any granulation tissue, bleeding, or pus. As a result, a decision was made to obturate tooth #41 with 26-04% gutta-percha points (Trunatomy confirm fit gutta percha point, Dentsply Sirona, USA), and tooth #31 was completely obturated using Bio-C Repair (Angelus Odontologia, Brazil), a root canal filling material. Following obturation, nano-hybrid composite (3M Z250 XT, 3M, USA) post-endodontic restorations were placed in teeth #31 and #41 (Figure 3, Panel B).

Six months after completing the treatment, the patient underwent an evaluation. Clinically, the patient did not have any symptoms, and the tissues surrounding the treated teeth were in a healthy condition. The periapical radiograph revealed no abnormalities and showed that the bone structure surrounding the roots had been fully restored (Figure 3, Panel C). These positive findings indicated the successful outcome of the treatment, with no signs of complications or recurrence of the previous issues.

## Discussion

Making a choice between endodontic treatment for a tooth with a bleak prognosis and extraction followed by implant placement can be difficult. Bell described internal resorption for the first time in 1830 [14] as a multifactor process linked to numerous factors, including systemic conditions, local factors, physiological resorption, and idiopathic resorption.

Internal resorption can usually be treated predictably because the process can be stopped by utilizing conventional root canal therapy to cut off the blood supply to the resorbing tissues. Remineralization of the defect may occur in situations with perforating flaws, resulting in the creation of hard tissue against which a permanent root canal filling can be compressed. However, in extreme circumstances, surgery or even an extraction may be necessary [3].

Owing to restrictions of MTA [15,16], bioceramic endodontic materials have been developed as substitute cement for repair. Bio-C Repair, in particular, being provided in a ready-to-use form (putty) has a benefit. It has a unique chemical composition and ultrastructural morphology, as well as good biocompatibility and positive effects on human dental pulp. On comparing Bio-C Repair to other biomaterials, which mostly contain calcium and oxygen, it was discovered that Bio-C Repair is primarily made of carbon (~35%) and oxygen (~34%), with a reduced percentage of calcium. Consequently, a stronger ability for tissue repair may be linked to this specific composition of Bio-C Repair. Comparative studies have shown that Bio-C Repair exhibits higher cell migration rates, adhesion, and viability compared to other bioceramics [13].

When dealing with bioceramics, it is important to note that they do not perform well in inflamed sites [17]. Therefore, the determination of the perforation limits and proper filling depends on minimizing excessive bleeding [18]. In the presented case, the perimeter of the perforation cavity was dried using paper tips, and bleeding was controlled with a diode-laser (Biolase epic x, Biolase, India) along with 5% sodium hypochlorite (Parcan, Septodont, India). Only then was the cavity filled with bioceramics (Bio-C Repair, Angelus Odontologia, Brazil). The use of a calcium hydroxide (RC Cal, Prime Dental, India) dressing before filling allowed for the neutralization of the environment and favored the characteristics of the bioceramics (Bio-C Repair, Angelus Odontologia, Brazil).

In these instances, the possible use of barriers to stop additional extrusion of the substance into the surrounding tissues [19] was not considered. Clinical examination and radiographic follow-up revealed that, despite the modest extrusion of the bioceramics (Bio-C Repair, Angelus Odontologia, Brazil), it did not appear to have an impact on the healing process.

Regarding the use of laser, the fundamental working principle of laser radiation is that the dissipated energy causes the selected tissue to respond as follows: it either causes the soft tissues to heat up and coagulate or heat up and vaporize. For numerous intraoral soft tissue surgical options, the laser is a practical substitute for the blade. Additionally, granulation tissue removal has been done using it. Among the many benefits, the main benefit of this therapy over traditional scalpel surgery is its effective hemostasis. It aids in the postoperative adaptation of normal connective tissue to the root surface by postponing epithelial migration on the root surface. It increases the likelihood of a new attachment in this way [20].

These findings support the use of bioceramics, such as Bio-C Repair, as an alternative to MTA in cases of extensive perforating internal resorption, considering their advantageous properties and potential for tissue repair.

## Conclusions

This case report demonstrates the successful non-surgical management of idiopathic extensive perforating internal root resorption using bioceramic materials, specifically Bio-C Repair. The use of bioceramics offers improved handling properties, biocompatibility, and potential tissue repair capabilities. The treatment protocol involved meticulous root canal disinfection, laser-assisted tissue debridement, and application of the bioceramic material. Clinical and radiographic follow-up confirmed the successful outcome with no symptoms and complete restoration of the surrounding bone structure. This case highlights the promising potential of bioceramics as an alternative to conventional materials for treating internal root resorption.
